# Understanding the Aggregation Mechanism of and Developing Stabilization Strategies for Recombinant Fibroblast Growth Factor 2

**DOI:** 10.3390/biom16060768

**Published:** 2026-05-23

**Authors:** Ruolan Cheng, Natalia Oganesyan, Andrew Lees, Igor A. Kaltashov

**Affiliations:** 1 Chemistry Department, University of Massachusetts-Amherst, Amherst, MA 01003, USA; ruolancheng@umass.edu; 2 Fina Biosolutions, Rockville, MD 20850, USA; natalia@finabio.com (N.O.);

**Keywords:** protein therapeutics, protein aggregation, native mass spectrometry, disulfide mapping, non-native disulfides, heparin binding proteins

## Abstract

Fibroblast Growth Factor 2 (FGF2) is a highly effective regulator of cell proliferation, differentiation, migration, and adhesion, suggesting a significant therapeutic potential as a tissue regeneration promoter both in acute and chronic tissue damage settings. Despite an extensive list of pathologies that lend themselves as viable targets for FGF2-based therapy (ranging from periodontics to burns to diabetic ulcers to coronary artery disease), the success record in the clinic remains modest, with no FDA approvals obtained so far. The inferior stability of this protein is frequently cited as the most significant factor behind its disappointing performance as a biotherapeutic. Multiple strategies have been designed and tested in an effort to ameliorate this problem, but the success remains elusive. We investigate the aggregation propensity of a recombinantly produced FGF2 using native mass spectrometry (MS) to identify conditions favoring formation of small soluble oligomers, which are considered precursors to larger aggregates. Tandem MS of proteolytic fragments produced by digestion of the oligomeric species allows the formation of external disulfide bonds to be identified as the process leading to oligomerization. Specifically, Cys-31 (one of the two unpaired cysteine residues in intact FGF2) appears to be a particularly active promoter of oligomerization by forming external disulfide bonds. As a high-pI protein, FGF2 readily associates with heparin, and molecular modeling identifies a positive charge basin proximal to Cys-31 as a potential heparin binding site, which can readily accommodate a synthetic heparin mimetic fondaparinux. Adding an equimolar amount of the latter to the FGF2 solution not only leads to formation of a stable protein/polyanion complex (as revealed by native MS), but also inhibits formation of FGF2 oligomers (presumably via a combination of steric hindrance and electrostatic repulsion). These findings advance our understanding of FGF2 stability, which will be invaluable for optimizing its formulation, storage, and administration.

## 1. Introduction

Protein aggregation remains one of the most serious challenges in the production, formulation, and storage of biopharmaceutical products. This keeps the aggregation propensity high on the list of critical quality attributes (CQA) of protein therapeutics across the entire spectrum of sizes, ranging from insulin to monoclonal antibodies [1]. Multiple techniques have been developed over the past two decades to detect and characterize both small oligomers and large aggregates of biopharmaceutical products [2,3,4], with electrospray ionization (ESI) mass spectrometry (MS) being undoubtedly one of the most critical components of this vast toolbox [5,6]. While the ability to detect the presence of oligomers and aggregates in protein therapeutic formulations is always at a premium, their structural characterization carries even more value. Indeed, the availability of detailed structural information on these elusive species may not only provide insight into their formation mechanism, but also offer rational strategies aiming at aggregation prevention. One intriguing feature that emerges from these studies is the important role played by cysteine side chains in triggering and directing protein oligomerization [7,8]. Indeed, the earlier ESI MS-based studies focused on direct monitoring of heat-triggered protein aggregation, all used proteins containing multiple cysteine residues [9,10]. Furthermore, the importance of cysteines as aggregation promoters was also noticed in the studies that used other means of inducing oligomerization of biopharmaceutical products [11,12]. This should not be surprising, as formation of external disulfide bonds (either through scrambling of the native disulfides or mispairing of free thiols) should provide a permanent lock for non-native (and, therefore, innately less stable) conformations. Therefore, transient disruption of the native structure (both covalent and higher-order) caused by various external factors (such as heat stress and pH variations among many others) may become permanently locked by way of formation of these external cross-links [13,14]. Irreversible accumulation of such “defects” will lead to the gradual growth of the aggregates, as has been observed in the earlier studies of the heat-induced aggregation of cysteine-rich proteins using a temperature-controlled ESI source [9]. While the general outlines of the aggregation mechanism are established [15,16,17], the ability to design strategies to minimize or indeed completely arrest the aggregation hinges upon the availability of a more nuanced and detailed picture of the initial steps of this process, namely formation of the small oligomeric species and identifying the specific cysteine residues that participate in these “aggregation seeding” events [18]. These tasks proved more challenging and can only be addressed on a case-by-case basis.

The protein examined in our work, fibroblast growth factor 2 (FGF2) is a highly effective regulator of cell proliferation, differentiation, migration, and adhesion [19,20,21]. Such impressive versatility suggests an enormous (but—regrettably—mostly untapped so far) therapeutic potential. The disappointingly modest success record of FGF2 as a biotherapeutic is largely due to the inferior stability of this protein, which manifests itself via partial unfolding [22], self-association, and even visible precipitation [23], giving rise to short in vivo half-life, reduced bioavailability, and immunological risks [24,25]. Even though multiple strategies have been tested over the past two decades to address this shortcoming, the success remains elusive [20,26]. We use a combination of size exclusion chromatography (SEC), native MS, and peptide mapping with LC/MS/MS to investigate the aggregation propensity of a recombinant form of FGF2 and identify the specific cysteine residue that is essential for the initial oligomerization steps. Careful consideration of the protein structure in the immediate vicinity of this aggregation trigger reveals the polycationic character of this patch and suggests that it is a likely binding site for polyanions, viz., heparin. Association of heparin or its derivatives with FGF2 proximal to this cysteine residue should inhibit formation of external disulfide bridges via a combination of steric hindrance and electrostatic repulsion, a proposition confirmed in our study with native MS.

## 2. Materials and Methods

### 2.1. Materials

The recombinant form of human FGF2 was produced in-house by Fina BioSolutions LLC (Rockville, MD, USA) using the Fina Gormet technology, and the purity was assessed with SDS-PAGE. The protein was kept in 20 mM HEPES, 0.5 M NaCl, 10% sucrose (*w*/*v*) (pH 7.7) at a concentration of 4 mg/mL. Fondaparinux and iodoacetamide (IAM) were purchased from Sigma-Aldrich Chemical Co. (St. Louis, MO, USA). The mass spectrometry grade Trypsin Gold was purchased from Promega (Madison, WI, USA). All other chemicals and solvents used in this work were of analytical grade or higher.

### 2.2. Methods

The extent of FGF2 oligomerization and aggregation was evaluated with size exclusion chromatography using a Phenomenex (Torrance, CA, USA) Yarra SEC-3000 column (3 µm, 7.8 × 300 mm) and an Agilent (Santa Clara, CA, USA) 1200 HPLC system. Isolation of a distinct oligomer fraction was accomplished under isocratic conditions (with 1.0 M ammonium acetate, pH 6.9 as the mobile phase to minimize electrostatic interaction of the polycationic FGF2 with the SEC matrix) at a flow rate of 0.8 mL/min. The collected fractions were concentrated and solvent-exchanged by ultracentrifugation (4000 rpm at 4 °C) using 10 kDa MW cutoff filters (Millipore Sigma, Burlington, MA, USA). The solvent-reconstituted samples were diluted to a final concentration of ca. 3–6 µM in 150 mM AmAc buffer prior to native MS measurements. All native MS measurements were carried out using a Synapt G2 HDMS (Waters, Milford, MA, USA) hybrid quadrupole/TOF mass spectrometer equipped with a nanospray source. The following ion source parameters were used to minimize dissociation of oligomers while maintaining acceptable sensitivity levels: sampling cone voltage, 190 V; trap DC bias, 2.2 V; ion source temp: 30 °C.

The intact-mass analysis of mass spectra of FGF2 under denaturing conditions was acquired with an on-line LC/MS^n^ system consisting of an HP 1100 (Agilent, Santa Clara, CA, USA) chromatograph and an LTQ mass spectrometer (Thermo Fisher Scientific, San Jose, CA, USA). A 5 μm, 4.6 mm × 50 mm C4 column (Grace VYDAC, Hesperia, CA, USA) was used for on-line separations with the following mobile phases: 0.1% formic acid in H_2_O (A) and 0.1% formic acid in acetonitrile (B), and the following linear gradient was employed: 5% B at 0 min to 95% B at 10 min with a flowrate of 0.2 mL/min. The ESI source parameters were set as follows: the spray voltage, 4.0 kV; the capillary voltage, 20 V; and the tube lens voltage, 120 V; the sheath, auxiliary, and sweep gas flows were set at 10, 10, and 0 arbitrary units, respectively; and the ion transfer tube temperature was maintained at 200 °C.

For the peptide mapping experiments, a stock solution of FGF2 was denatured in 8 M urea at room temperature for 10 min, followed by addition of 50 mM ammonium bicarbonate to dilute the urea to a final concentration of 0.8 M. A 6 μL aliquot of 100 mM IAM was then added to the denatured and diluted FGF2 solution and incubated at room temperature in the dark for 30 min. The proteolytic digestion was performed by adding trypsin at an enzyme-to-substrate ratio of 1:20 (*w*/*w*), followed by incubation at 37 °C overnight. The tryptic digests were analyzed with the LC/MS^n^ system described above, with one exception: the C4 column was substituted with a C18 column (a 3.5 μm, 2.1 × 150 mm 300Extend column, Agilent, Santa Clara, CA, USA), and the column temperature was maintained at 40 °C. The mobile phases were the same as those employed for the intact-mass analysis, but the linear gradient ramp (from 5% to 95% B) was extended to 60 min (the flow rate was maintained at 0.2 mL/min). The total peptide mass injected in a single experiment varied between 3.0 and 3.5 μg. The ESI source parameters were set as follows: spray voltage, capillary voltage, and tube lens voltage were 4.5 kV, 29 V and 60 V, respectively; sheath, auxiliary, and sweep gas flows were set to 30, 0, and 5 arbitrary units, respectively; and the ion transfer tube temperature was maintained at 275 °C. The precursor ion selection for the on-line MS/MS analyses was carried out in a data-dependent acquisition (DDA) mode using collision-induced dissociation (CID) with a normalized collision energy of 35. Data processing was carried out using built-in Xcalibur (Version 4.2.47) (Thermo Fisher Scientific, Waltham, MA, USA).

All molecular modeling work was performed with the Maestro platform V13.3 (Schrödinger Release 2022-3, Schrödinger LLC, New York, NY, USA) using the OPLS4 force field. The initial structure of FGF2 was taken from the crystal structure (PDB: 2M49). The 3D structure of the heparin mimetic fondaparinux was extracted from PDB 4R9Y. The 100 ns MD simulations of FGF2/fondaparinux complexes were set up using a neutralized system in explicit water and 150 mM NaCl at 310 K.

## 3. Results

**Figure 1:**
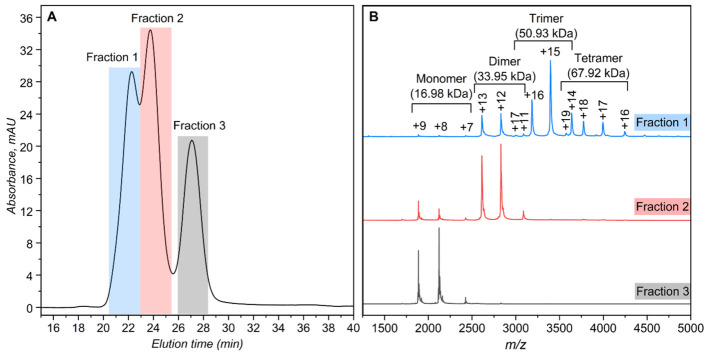
SEC analysis of FGF2 stock solution (**A**) and native MS analysis of the three SEC fractions (**B**).

**Figure 2:**
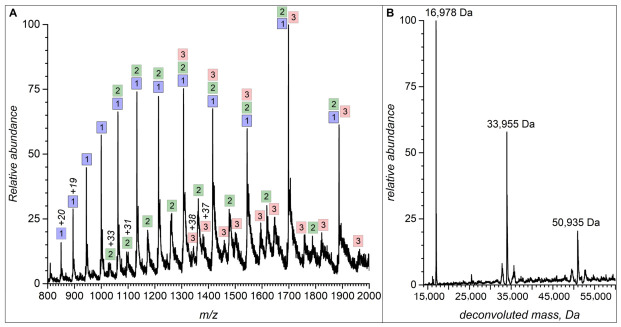
MS analysis of the FGF2 under denaturing conditions. Numbers 1, 2, and 3 in the filled squares indicate the FGF2 monomers, dimers, and trimers, respectively (**A**) and a deconvoluted mass distribution (**B**).

The post-production testing of FGF2 with SDS-PAGE reveals a low-intensity band in the region corresponding to a molecular weight exceeding that of FGF2 by ca. two-fold (see Figure S1 in Supporting Information). Re-evaluation of the same sample with SEC following a one-week-long storage at −20 °C (carried out at high salt (1.0 M) to suppress the secondary electrostatic interactions of the polycationic protein with the SEC resin) revealed the presence of at least three different components with significant mass differences (Figure 1A). Although the three peaks are not baseline-resolved, the corresponding fractions have been collected and their contents analyzed with native MS. The resulting mass spectra provide a clear indication that the early-eluting species are FGF2 dimers, trimers, and tetramers, while the latest eluting species is the monomeric form of the protein (Figure 1B). In order to determine the nature of the interactions giving rise to the small oligomers (non-covalent clamping vs. formation of external disulfide linkages), the entire stock solution was diluted in a denaturing solvent (0.1% aqueous formic acid), and the intact-mass analysis was carried out with reversed-phase LC/MS. The resulting mass spectrum averaged across the entire elution window contains distinct signals of several different ionic species (Figure 2A), and its deconvolution indicates that there are three components contributing to the total ionic signal in the mass spectrum (Figure 2B). The masses of these components match those of the FGF2 monomer (16,978 ± 1 Da), as well as its dimer (33,955 ± 13 Da), and trimer (50,935 ± 20 Da). These observations indicate that the FGF2 oligomers are covalently linked, most likely through intermolecular disulfide bonds.

**Figure 3:**
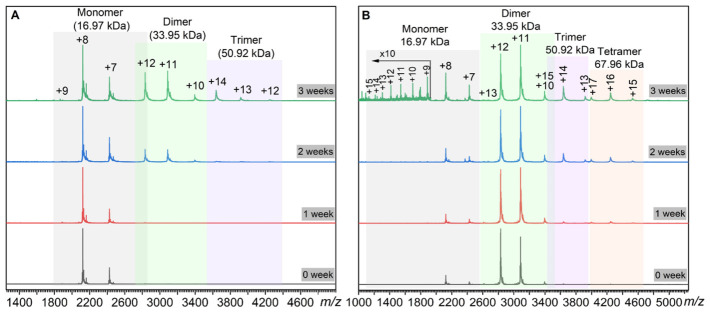
Stability of SEC-fractionated FGF2 monomers (**A**) and dimers (**B**) in 150 mM ammonium acetate at 4 °C over the course of three weeks monitored with native MS.

The FGF2 oligomerization propensity was further tested by incubating the SEC-purified monomeric species in ammonium acetate at physiological pH (6.9) and ionic strength (0.15 M) maintained at 4 °C and analyzing the protein oligomerization state with native MS (Figure 3). Although no signals of FGF2 oligomerization were evident in the mass spectrum acquired after a week-long storage, more extended protein exposure to ammonium acetate gives rise to dimer formation (visible after two weeks) and trimers (after three weeks) (Figure 3A). Similar stability studies were carried out with the SEC-purified FGF2 dimers (Figure 3B). Consistent with the FGF2 monomer behavior, the dimer remains stable for at least a week-long exposure to ammonium acetate at low temperature, followed by the appearance of higher-order oligomers at longer storage periods.

**Figure 4:**
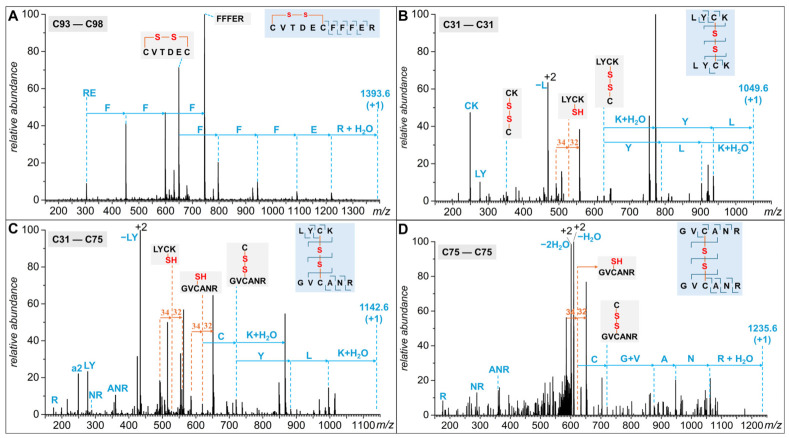
Detection of native (**A**) and non-native (**B**–**D**) disulfides in protein species present in the FGF2 stock solution by LC/MS/MS analysis of a tryptic digest of an aliquot of the stock solution.

The covalent nature of the subunit inter-connection in the small oligomers observed under denaturing conditions is further confirmed by peptide mapping carried out using the FGF2 stock solution, following its treatment with iodoacetamide (to alkylate the free thiol residues) prior to the proteolytic step. Since the tryptic digestion was not preceded by the disulfide reduction, there is a prominent signal corresponding to a tryptic fragment C93–R103 containing the internal (native) disulfide bond C93–C98 (see Figure S3 in Supporting Information). This peptide’s identity is established based on the results of the on-line MS/MS measurements that reveal the presence of the FFFER segment via *b*- and *y*-fragment ladders (Figure 4A). The termination of both ladders at C98 (no fragments are present within the C93–C98 segment of the peptide) and the 2 Da ionic mass reduction compared to the sequence-based peptide mass calculation indicate that the C93–C98 disulfide is intact. In addition to the native C93–C98 disulfide, the MS/MS measurements reveal the presence of non-native thiol–thiol connections: C31–C31, C31–C75, and (to a significantly lesser extent) C75–C75 (Figure 4B–D and Figure S3).

**Figure 5:**
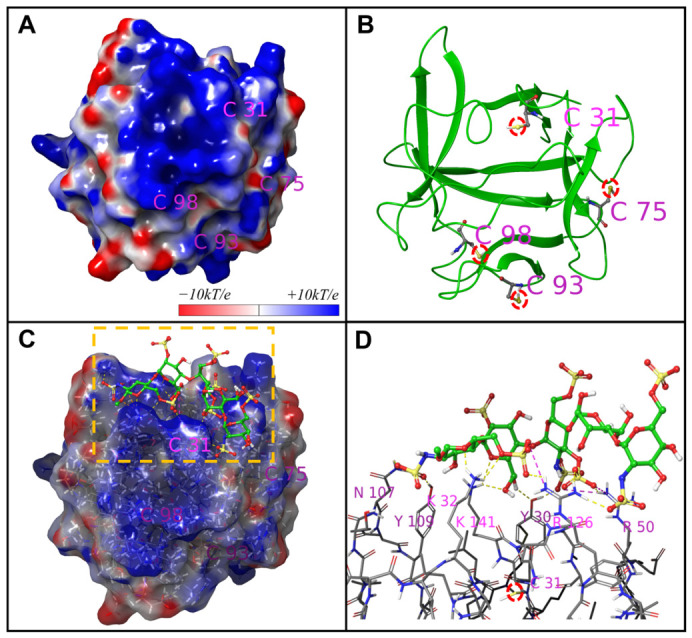
Electrostatic potential distribution of the surface of FGF2 (PDB id 2M49) (**A**), Cys residue distribution on FGF2 (**B**), molecular modeling of the FGF2/fondaparinux interactions (**C**), and the detailed binding interface of FGF2/fondaparinux (yellow dashed box) showing salt bridges and hydrogen bonds with the purple and yellow dashed lines, respectively (**D**).

**Figure 6:**
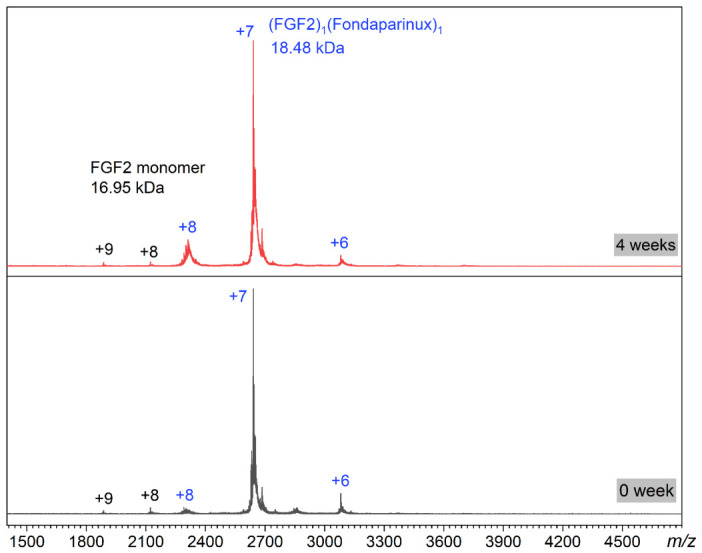
Native MS of an equimolar mixture of the SEC-purified FGF2 monomer and fondaparinux. The FGF2 and fondaparinux mixing solution was detected at the beginning (**bottom**), and after four-week storage (**top**) in 150 mM ammonium acetate at 4 °C. The absolute signal intensities are nearly equal to each other, indicating no detectable protein loss due to precipitation during the storage (see Supplementary Information for more detail).

The distribution of cysteine residues on the FGF2 surface indicates that the shortest spatial separation occurs between C93 and C98 by measuring the length between two sulfur atoms using the Maestro software suite (Figure 5B). Notably, both residues are positioned within a loop segment, suggesting that their proximity and structural context facilitate the formation of a native disulfide bond following synthesis. Examination of the FGF2 electrostatic potential surface reveals another notable feature in the immediate environment of this residue: a pronounced local concentration of positive charge surrounding C31 (Figure 5A). Such regions are critical for mediating interactions with negatively charged ligands, most notably heparin and heparan sulfate proteoglycans. Indeed, the native MS analysis of SEC-purified FGF2 monomers incubated with a polyanionic pentasaccharide fondaparinux (a small synthetic mimic of heparin) [27] at an equal molar ratio reveals strong binding (Figure 6, bottom), with only two species present: a 1:1 FGF2/fondaparinux complex (major) and free FGF2 (minor). After four weeks under identical storage conditions (physiological pH, ionic strength, and 4 °C), the native mass spectrum of this FGF2/fondaparinux mixture solution remains essentially unchanged and closely overlaps with the initial spectrum, indicating no detectable FGF2 oligomerization (Figure 6, top).

## 4. Discussion

Protein aggregation, along with its induced immunogenicity, continues to represent a major obstacle in the development and deployment of recombinant protein therapeutics [28,29]. Elucidating the underlying molecular mechanisms that govern aggregation, as well as assessing its impact on biological potency, is essential for the rational design of effective mitigation strategies. Mass spectrometry (MS) coupled with size exclusion chromatography (SEC) has emerged as a powerful analytical platform for the identification, quantification, and structural characterization of protein aggregates [30,31]. Given its ability to provide detailed insight into aggregation profiles, this approach plays a critical role in ensuring the quality and consistency of biotherapeutic products. By examining the abundance distribution of the monomeric and oligomeric species in Figure 1A (SEC) and Figure 2B (deconvoluted mass), one thing that is immediately noticed is that the monotonic decline of the ionic abundance in the deconvoluted spectrum (monomer > dimer > trimer) does not recapitulate the abundance distribution in the SEC diagram. This apparent incongruence is a result of the chromatographic data reflecting weight concentrations, while the MS data reflect molar concentrations (with additional bias against higher-mass species) as discussed by Wang et al. [32] Once this is taken into account, and the MS-based distribution adjusted to reflect the mass concentrations, the two distributions agree remarkably well (Figure 2B).

The persistence of FGF2 oligomers under denaturing conditions (vide supra) hints at their covalent nature, but cannot be used as a single definitive piece of evidence supporting the notion of protein oligomerization driven by formation of the external disulfide bonds. Although thiol oxidation does result in a protein mass shift, the mass measurement precision afforded by the ion trap mass analyzer is not sufficient for distinguishing non-covalently bound oligomers form their disulfide-linked counterparts (formation of a single disulfide bond reduces the protein mass by 2.016 Da, and detection of this mass shift would require mass resolution exceeding 15,000, which unfortunately is not feasible with the ion trap instrument used in this work). Nevertheless, the appearance of the mass spectrum is consistent with the notion of both the dimeric and trimeric forms of FGF2 being covalently linked oligomers (Figure 2A). The nearly identical charge densities displayed by the monomeric form of FGF2 and the small oligomers indicate that the latter are in the unfolded states as well, but do not dissociate into monomeric units, which implies that the monomeric units are held together by covalent (rather than non-covalent) interactions. Indeed, their charge state distributions nearly mirror that of the FGF2 monomer (which is bimodal, consistent with the protein denaturation without the complete loss of all higher order structure elements [33]).

During the assessment of the FGF2 oligomerization propensity (Figure 3), our choice of ammonium acetate was driven by an intent to subject FGF2 to a stress study, by slightly increasing the conformational dynamics of the protein (to promote enhanced solvent exposure of its hydrophobic elements and/or free cysteine residues) without compromising the overall native fold. Indeed, despite being a common electrolyte system used in native MS and offering significant advantages over ammonium bicarbonate [34], ammonium acetate has been shown to exert a subtle influence on the protein higher-order structure that is not observed in physiologically relevant electrolytes, such as NaCl [35]. Therefore, it lends itself as a perfect agent for the stress stability studies aimed at exploring the early stages of FGF2 oligomerization/aggregation by enhancing the conformational “fluidity” of the protein.

Disulfide bond mapping by MS/MS analysis identified one native disulfide bond (C93–C98) and three non-native disulfide linkages (C31–C31, C31–C75, and C75–C75) (Figure 4). While it is possible to argue that the C31–C75 disulfide bond might be internal, its formation would require a significant distortion of the monomeric FGF2 structure, and since no signs of the monomeric species denaturation are visible in the native MS of the FGF2 stock solution (see Figure S2 in Supporting Information), one must conclude that all three non-native disulfide configurations are specific to the oligomeric forms of FGF2. Not surprisingly, the abundance of the peptide ion containing the native disulfide bond C93–C98 dwarfs all other disulfide-containing ions, while the non-native disulfide C75–C75 gives rise to the lowest-abundance signal (see Figure S3 in Supporting Information). The non-native C31–C31 and C31–C75 pairings give rise to peptide dimer ions that are nearly equiabundant. However, we note that the primary structure of the latter is expected to favor a stronger response in ESI due to the presence of an arginine residue and a mixture of hydrophobic and polar side chains that typically enhance the ESI response due to the preferred positioning of such peptides at the water/air interface (i.e., at the surface of the electrosprayed droplets) [36]. Therefore, despite the near-parity of the ionic abundance of the peptides representing the peptide dimers connected by non-native C31–C31 and C31–C75 disulfide bonds, the former is expected to have a significantly larger relative concentration in solution.

Since C31 appears to be a critical structural element of FGF2 acting as the primary driver of its oligomerization, the protein surface structure in the vicinity of this residue has been examined carefully using molecular modeling tools. This residue is indeed positioned on the protein surface, suggesting that its side chain can interact with other free thiol groups without the need for protein denaturation (Figure 5B). Another intriguing feature of the immediate environment of this residue revealed by the examination of the FGF2 conformation is a significant local density of the positive charge (the +10*k*T/*e* equipotential surface covering an area of ca. 360 Å^2^ in the immediate vicinity of C31 (Figure 5A)). This observation suggests a possible strategy for attenuating the reactivity of this free cysteine residue, namely complexation with polyanions, such as heparin derivatives. The latter has been shown to form stable complexes with other FGFs via electrostatic interactions [37,38], and the strong polycationic character of FGF2 all but guarantees that it is also a strong heparin binder. Indeed, the native MS analysis of SEC-purified FGF2 monomers incubated with a polyanionic pentasaccharide fondaparinux reveals strong binding (Figure 6), consistent with the earlier studies reporting effective association of this polyanion with other polycationic proteins [39,40].

This makes the “bridging” configuration thermodynamically unfavorable (by depriving at least a few sulfate groups within the pentasaccharide chain of the “pairing” positive charges). Similar behavior (a strong preference for accommodation of the entire polyanion chain) has been observed previously for another small protein with an extended positive charge basin, platelet factor 4 [41]. A tight binding of a polyanionic chain to the FGF2 surface proximal to C31 should have a negative effect on the ability of this residue to participate in the formation of external disulfide bonds due to the steric hindrance effect (as well as strong electrostatic repulsion between the two polyanionic chains). Since formation of the external disulfide bonds (in particular, those involving C31) is critical for FGF2 oligomerization (*vide supra*), fondaparinux association with the protein in the vicinity of this critical residue should arrest the oligomerization process by introducing steric hindrance and electrostatic repulsion.

These observations confirm the modeling-informed hypothesis that FGF2 association with fondaparinux attenuates the reactivity of the free thiol at C31 very dramatically. Furthermore, they provide unequivocal evidence that FGF2 oligomerization can be effectively and completely arrested by fondaparinux without requiring large concentrations of the latter. This unique property of fondaparinux makes it attractive as a potential stability-enhancing excipient. Indeed, fondaparinux has been developed as a safe alternative to heparin (unlike heparin, it does not precipitate heparin-induced thrombocytopenia, or HIT [42]). Furthermore, being rather small in size (its physical dimensions do not exceed 22 Å even in the fully extended conformation), fondaparinux is expected to undergo rapid clearance via glomerular filters, further minimizing the risk of adverse side effects (such as internal bleeding commonly associated with administration of the blood-thinning medicines).

## 5. Conclusions

The recombinantly produced FGF2 has high aggregation propensity, reflecting the inferior stability characteristic of many proteins within the FGF family, but the specific details of the molecular mechanism underlying aggregation (especially the early steps of this process) remain to be clarified. A combined use of SEC, native MS, reversed-phase LC/MS, and peptide mapping with LC/MS/MS detection identify formation of non-native external disulfide bonds as the dominant mechanism driving formation of small protein oligomers (dimers to tetramers). Of the two free cysteine residues within FGF2, C31 appears to be a particularly active promoter of the protein’s oligomerization. Inspection of the electrostatic potential distribution at the FGF2 surface identifies an extended basin of positive charge localized within the immediate vicinity of C31. The presence of this positive charge basin should enable capable of accommodating polyanions, such as heparin fragments, and this association may result in a steric hindrance interfering with the formation of external disulfide bridges. Indeed, incubation of purified FGF2 monomers with a heparin mimetic fondaparinux not only leads to a stable protein/polyanion association but also effectively inhibits the formation of FGF2 oligomers. Therefore, elucidation of the molecular mechanism of the early aggregation stages of this protein not only advances our understanding of FGF2 stability, but also provides invaluable information for optimizing its formulation, storage and administration. Future work will assess the long-term stability of FGF2 under various storage conditions, including different temperatures, pHs, and buffer compositions. Furthermore, the potential impact of heparin as an attenuator of the FGF2 therapeutic properties must be carefully evaluated.

## Data Availability

Data are contained within the article and Supplementary Materials.

## Supplementary Materials

The following supporting information can be downloaded at: https://www.mdpi.com/article/10.3390/biom16060768/s1, Figure S1: SDS-PAGE gel analysis of FGF2 stock solution without and with Dithiothreitol (DTT); Figure S2: Native MS analysis of FGF2 stock solution; Figure S3: Extracted ion chromatogram of four different disulfide bond linkages in FGF2 stock solution obtained by LC–MS/MS; Figure S4: Raw data for Figure 6 of the paper showing the invariance of the absolute intensity of the ionic signal.
