# Supplementary material for: Understanding the Aggregation Mechanism of and Developing Stabilization Strategies for Recombinant Fibroblast Growth Factor 2
Source: Biomolecules. 2026 May 23;16(6):768. doi: 10.3390/biom16060768 (PMC13297325; doi:10.3390/biom16060768)

Supplementary Information for

Understanding the Aggregation Mechanism and Developing Stabilization Strategies for the Recombinant Fibroblast Growth Factor 2

Ruolan Cheng,[1] Natalia Oganesyan,[2] Andrew Lees,[2] and Igor A. Kaltashov[1,*]

1 *Chemistry Department, University of Massachusetts-Amherst, Amherst, MA*

2 *Fina Biosolutions, Rockville, MD*

* *Correspondence: Address correspondence to: Igor A. Kaltashov, 240 Thatcher Road, Life Sciences Laboratories N369, Amherst, MA 01003. Email: kaltasho@umass.edu*

Table of Contents

Figure S1. SDS-PAGE gel analysis of FGF2 stock solution without and with Dithiothreitol (DTT).

Figure S2. Native MS analysis of FGF2 stock solution.

Figure S3. Extracted ion chromatogram of four different disulfide bond linkages in FGF2 stock solution obtained by LC–MS/MS.

Figure S4. Raw data for Figure 6 of the paper showing the invariance of the absolute intensity of the ionic signal.

[*] Address correspondence to: Igor A. Kaltashov, 240 Thatcher Road, Life Sciences Laboratories N369, Amherst MA 01003. Email: kaltasho@umass.edu

Figure S1. SDS-PAGE gel analysis of FGF2 stock solution without and with Dithiothreitol (DTT), corresponding to the middle line and right line, respectively.

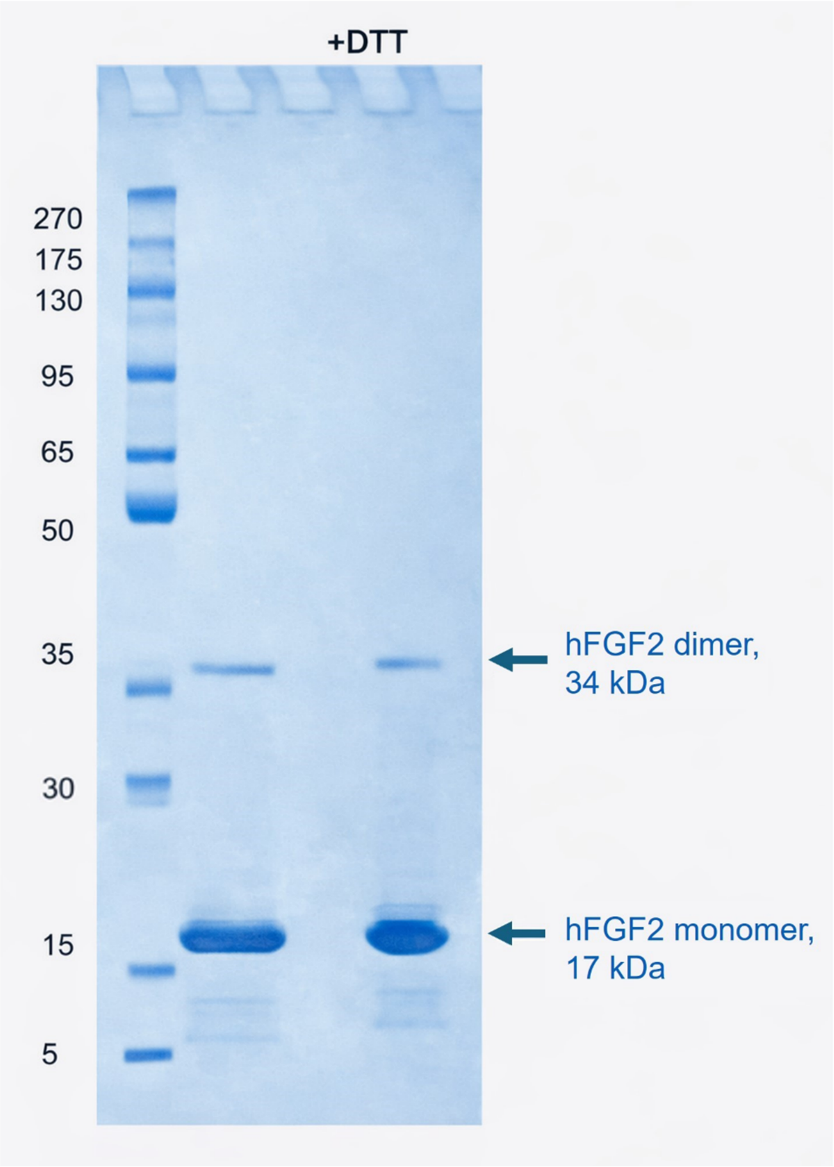

Figure S2. Native MS analysis of FGF2 stock solution.

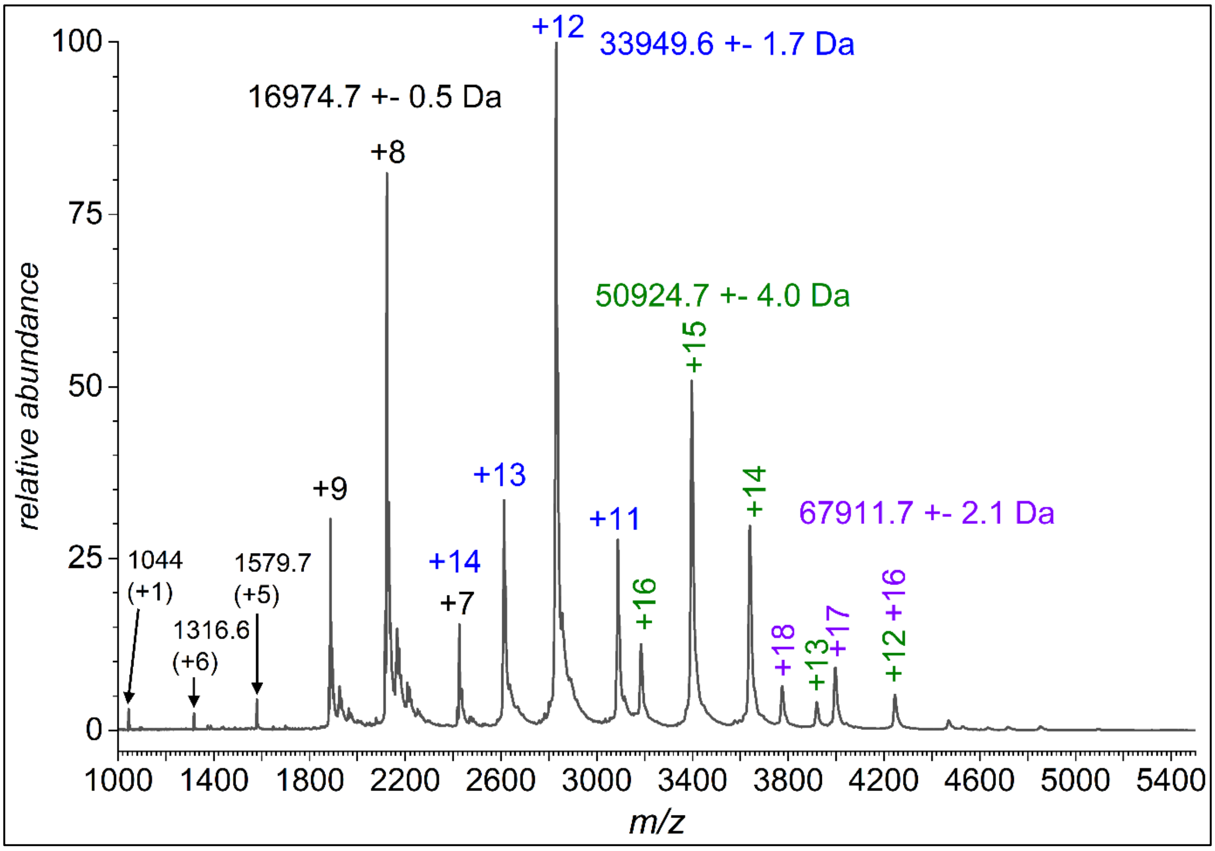

Figure S3. Extracted ion chromatogram of four different disulfide bond linkages in FGF2 stock solution obtained by LC–MS/MS.

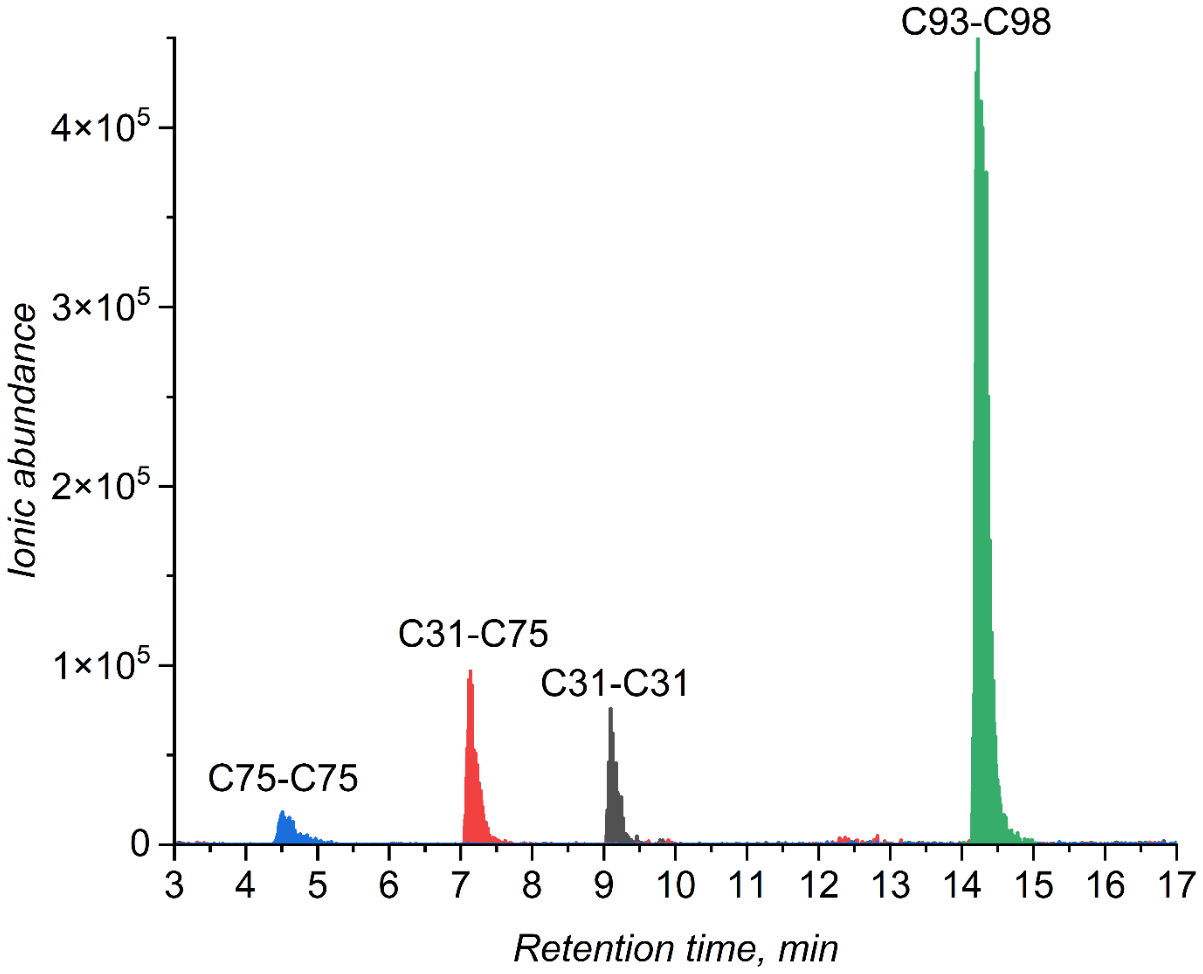

Figure S4. Raw data for the Figure 6 of the paper (Native MS of an equimolar mixture of the SEC-purified FGF2 monomer and fondaparinux) showing the invariance of the absolute intensity of the ionic signal, indicating that no loss of the protein material has occurred in solution over the four-week period.

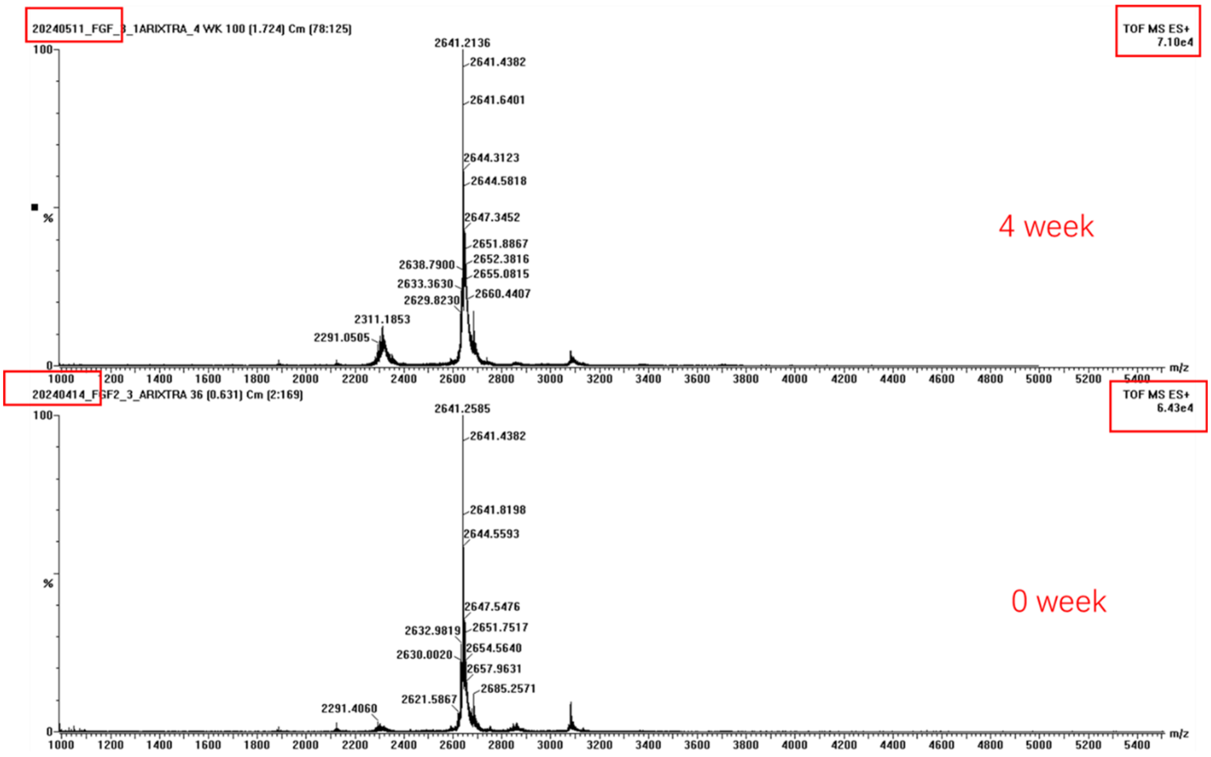

Supplement: Supplementary file 1 [file biomolecules-16-00768-s001.zip › biomolecules-4263521-supplementary.pdf]
